# Smart Internal Bio‐Glues

**DOI:** 10.1002/advs.202203587

**Published:** 2022-07-28

**Authors:** Hengjie Zhang, Jianhua Zhang, Xu Peng, Zhan Li, Wanjie Bai, Tianyou Wang, Zhipeng Gu, Yiwen Li

**Affiliations:** ^1^ College of Polymer Science and Engineering State Key Laboratory of Polymer Materials Engineering Sichuan University Chengdu 610065 China; ^2^ Experimental and Research Animal Institute Sichuan University Chengdu 610041 China

**Keywords:** adhesion, hemostasis, internal wound healing, smart bio‐glues

## Abstract

Although smart bio‐glues have been well documented, the development of internal bio‐glues for non‐invasive or minimally invasive surgery is still met with profound challenges such as safety risk and the lack of deep tissue penetration stimuli for internal usage. Herein, a series of smart internal bio‐glues are developed via the integration of o‐nitrobenzene modified biopolymers with up‐conversion nanoparticles (UCNPs). Upon irradiation by near‐infrared (NIR) light, the prepared smart bio‐glues can undergo a gelation process, which may further induce strong adhesion between tissues under both dry and wet conditions based on multi‐interactions. Moreover, those NIR light‐responsive bio‐glues with deeper tissue penetration ability demonstrate good biocompatibility, excellent hemostatic performance, and the potent ability to accelerate wound healing for both external and internal wounds. This work provides new opportunities for minimally invasive surgery, especially in internal wound healing using smart and robust bio‐glues.

## Graphical abstract

The smart internal bio‐glues are developed via the integration of o‐nitrobenzene modified biopolymers with up‐conversion nanoparticles. Upon near‐infrared light irradiation, those bio‐glues demonstrate strong adhesion, good biocompatibility, excellent hemostatic performance, and accelerated wound healing for both external and internal wounds. This work provides new opportunities for minimally invasive surgery and internal wound healing using smart bio‐glues.

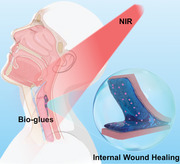

## 1 Introduction

Bio‐glues have attracted tremendous interest during recent decades for surgical applications owing to their promising physicochemical and biological properties, including strong bioadhesion, rapid hemostasis, low cost, and good biocompatibility.^[^ ^1^, ^2^, ^3^, ^4^, ^5^, ^6^, ^7^, ^8^, ^9^, ^10^, ^11^, ^12^, ^13^ ^]^ Smart bio‐glues for wound healing, tissue adhesion, and other biomedical applications are particularly interesting by exhibiting stimuli‐responsive features compared with conventional samples.^[^ ^14^, ^15^, ^16^, ^17^, ^18^, ^19^, ^20^ ^]^ For example, light‐responsive bio‐glues with contact‐free, spatiotemporal regulation, rigorous tailor, remote control, and other unique advantages have been widely used in wound healing,^[^ ^21^ ^]^ adhesive hemostasis,^[^ ^22^ ^]^ and tissue adhesion.^[^ ^23^, ^24^, ^25^ ^]^ Several kinds of smart polymers would undergo a sol‐gel transition process upon irradiation by UV or visible light, which could achieve rapid hemostasis, biomimetic tissue engineering, and tissue sealing.^[^ ^26^, ^27^, ^28^, ^29^ ^]^ Although many of their efforts have been well documented so far, the exploration of internal bio‐glues for non‐invasive or minimally invasive surgery has still met with profound challenges such as safety risk and the lack of deep tissue penetration stimuli for internal usage.^[^ ^3^ ^0–^ ^32^ ^]^ It is still highly important and urgently needed nowadays to address this issue by fabricating a new kind of smart bio‐glues for internal wound healing and tissue adhesion.

Near‐infrared (NIR) light has received great attention in internal and implantable biomedical materials due to its excellent tissue penetration depth, high stability, and low energy dosage.^[^ ^33^, ^34^, ^35^ ^]^ Recently, NIR light‐triggered hydrogels have been widely studied for photothermal therapy, tissue repair, drug release, and cell engineering, and the security concerns and thermal side effects have been demonstrated in several kinds of animal models.^[^ ^35^, ^36^, ^37^, ^38^ ^]^ Notably, up‐conversion nanoparticles (UCNPs)‐assisted light‐triggered reactions as a NIR light‐responsive system, could easily convert NIR light into UV light as an internally secondary source to induce kinds of photochemical reactions,^[^ ^39^, ^40^, ^41^ ^]^ which have been used for in vivo 3D printing^[^ ^42^ ^]^ and controlled drug release.^[^ ^41^ ^]^ Therefore, we speculated that one possible solution for the development of smart internal bio‐glues for non‐invasive or minimally invasive surgery should focus on the NIR light‐responsive systems used in deep tissues by the incorporation of highly efficient UCNPs.

**Figure 1:**
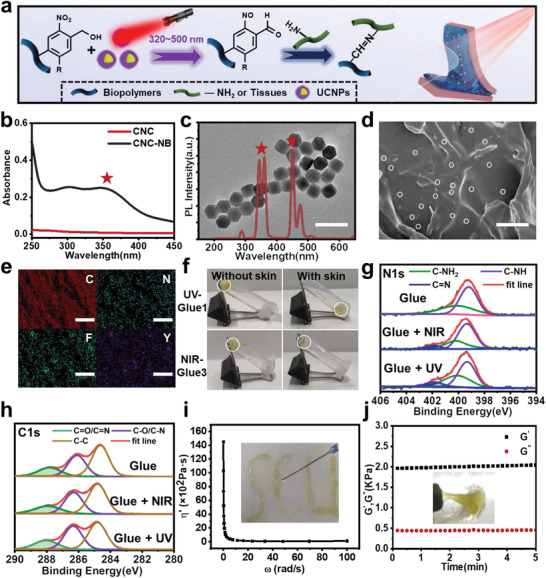
Preparation and characterization of smart bio‐glues. a) Schematic illustration of smart bio‐glues. b) UV–vis spectra of CNC‐NB and CNC in deionized water. c) TEM image and up‐conversion luminescence emission spectra of UCNPs. (Scale bar: 100 nm) d) SEM image of Glue 3. The white circles showed the uniform distribution of UCNPs. (Scale bar: 500 nm) e) EELS mapping analysis of Glue 3. (Scale bar: 25 µm) f) Experimental results of light‐triggered hydrogels formation without and with pigskin‐covered. The white circles represent the gel state. g) N 1s peaks and h) C 1s peaks in XPS spectra of bio‐glues without light, with UV, and NIR in 2 min, respectively (C=O/C=N indicates C=O or C=N). i) Shear‐thinning of Glue 3 after NIR irradiation. The inset showed the photograph of Glue 3 extruded in medical needles. j) Dynamic storage modulus (G′) and loss modulus (G″) at different time points of Glue 3 after NIR irradiation. The inset showed the photograph of Glue 3 after NIR irradiation.

Herein, we reported our effort towards this goal through the facile fabrication of smart internal bio‐glues consisting of UCNPs and o‐nitrobenzyl alcohol (NB) modified biopolymers (NB‐biopolymers) (**Figure** **1**a). The UCNPs within the glues could efficiently convert NIR light into UV light as an internally secondary light source to induce the imine crosslinking reaction in vivo, which could subsequently crosslink onto tissue surface. These NIR light‐responsive bio‐glues with deeper tissue penetration ability and lower safety risk can perform their bioadhesive properties in internal wound healing without suturing under minimal invasive treatment. This work could provide new opportunities for deeper internal tissue adhesion and non‐invasive or minimally invasive surgery by using smart and robust bio‐glues.

## 2 Results and Discussion

### 2.1 Preparation and Characterization of Bio‐Glues

To construct the smart internal bio‐glues, NB‐modified biopolymers (i.e., NB‐CNC, NB‐CMC, NB‐HA, NB‐SA, NB‐PAA, NB‐Gel, NB‐COL) and UCNPs (i.e., *β*‐NaYF_4_: 30% Yb^3+^ / 0.3% Tm^3+^as the typical example) were prepared according to previous studies.^[^ ^42^, ^43^, ^44^ ^]^ The synthesis processes and characterization of NB and NB‐biopolymer were shown in Figures S1–S4, Supporting Information. In particular, NB‐CNC with the highest NB grafting ratio (≈8.33%) was selected as the research model to further fabricate smart bio‐glues. Additionally, the aqueous lanthanide‐doped UCNPs with a uniform size of ≈50 nm and great up‐conversion efficiency at ≈365 and ≈450 nm region was also successfully prepared (Figure S5, Supporting Information), which could be easily incorporated into the bio‐glues via kinds of non‐covalent interactions with the polymer chains, such as molecular assembly, hydrogen bond, and electrostatic interactions.^[^ ^39^, ^42^, ^45^ ^]^ Notably, the typical emission bands of UCNPs had a great overlap with the structural transition absorption band of NB (≈320–500 nm) (Figure 1b,c), which suggested that these UCNPs incorporated NB‐biopolymer bio‐glues could perform their adhesive behavior by converting NIR light.

Then, a series of bio‐glues were prepared by mixing UCNPs (0%, 0.5%, and 1% w/v) with 60 mg mL^−1^ NB‐CNC and amine‐functionalized carboxymethyl chitosan (CMC) aqueous solution, which were named as Glue *i* (*i* = 1 to 3), respectively (Table S1, Supporting Information). As shown in Figure 1d, the existence of UCNPs could be clearly observed in Glue 3. In addition, the electron energy‐loss spectroscopy (EELS) elemental mapping images of Glue 3 further demonstrated that the C, O, N, F, Y, Na, Tm, and Yb elements were all uniformly distributed (Figure 1e and Figure S6a,b, Supporting Information). As demonstrated in an X‐ray photoelectron spectroscopy (XPS) survey result (Figure S6c, Supporting Information), we also found the additional appearance of F and Y elements within Glue 3 compared to Glue 1. All those evidence suggested the uniform distribution of UCNPs within the bio‐glues.

In addition, the sol‐gel transition behaviors irradiated by UV and NIR light were then evaluated without and with a pigskin. As expected in Figure S6d, Supporting Information, it was observed that Glue 3 could form a hydrogel without a covered pigskin similar to Glue 1. Additionally, Glue 3 could also undergo similar transition processes to form a hydrogel with a covered pigskin, but Glue 1 didn't work covered with a thin pigskin after UV irradiation, which indicated that the internal usage of these smart bio‐glues consisting of UCNPs (Figure 1f and Figure S6e, Supporting Information). Notably, we could achieve the optimized effect of sol‐gel transition and tissue adhesion for thicker tissue by adjusting the parameters including UCNP concentrations, NIR power, and irradiation time (Figure S6f, Supporting Information). For example, we could increase the UCNPs concentrations instead of increasing NIR power and irradiation time to achieve a better adhesion effect and minimize the thermal side effect. To further investigate the gel formation mechanism under NIR irradiation, we compared the XPS spectra of glues without light, with UV and NIR light, respectively. As shown in Figure 1g, the characteristic peaks of C−NH_2_ (400.07 eV) and C–NH (399.2 eV) were assigned to CMC and CNC‐NB. However, the emergence of the C=N (401.8 eV) peak could be observed after UV and NIR light irradiation, demonstrating the appearance of imine crosslinking in hydrogels. Note that the content of C=N produced under NIR irradiation was similar to the one generated by UV irradiation at the same time, while the bio‐glues without light irradiation only contained C=O, which further demonstrated that the formation of imine bonds may play vital roles in hydrogel crosslinking (Figure 1g,h).

Based on various kinds of non‐covalent and covalent multi‐interactions within bio‐glues, it was observed that Glue 3 could be easily extruded in medical needles and also perform the shear thinning property after NIR irradiation, which was favored for the minimally invasive tissue sealing and clinical injection applications in vivo (Figure 1i). Additionally, the dynamic rheological behaviors of each bio‐glue after irradiation have been performed at 25 °C, and it was found that the dynamic storage modulus (G′) of glues was greater than the loss modulus (G′′), which proved the sol‐gel transition after light irradiation (Figure 1j). Notably, the G′ increased from 0.52 to 1.96 KPa from Glue 2 to Glue 3, which indicated the modulus of bio‐glues after NIR irradiation was increasing with the increase of UCNPs content (Figure S6g,h, Supporting Information). And the storage modulus of Glue 3 was similar to Glue 1 after UV irradiation, which proved that bio‐glues with sufficient UCNP concentrations after NIR irradiation could achieve the similar effect of UV‐triggered glues.^[^ ^43^, ^44^ ^]^ Furthermore, the swelling ratio of bio‐gels was usually quite low when used for a short time, and they still maintained intact structure after fully swelling for a long time. For instance, the swelling ratio of Glue 3 after NIR irradiation was recorded as ≈150% and ≈300% at 6 and 60 h, respectively (Figure S6i, Supporting Information), which is in line with previous reports.^[^ ^44^, ^46^ ^]^ Moreover, the porous structures from scanning electron microscopy (SEM) images would benefit the hemostasis applications, which suggested the hydrogels could maintain good stability even after swelling with blood in vivo. (Figure S6j, Supporting Information). Together, these results encouraged us to further employ those smart bio‐glues for tissue adhesion and wound healing in vivo.

### 2.2 Adhesion Capability of Bio‐Glues

**Figure 2:**
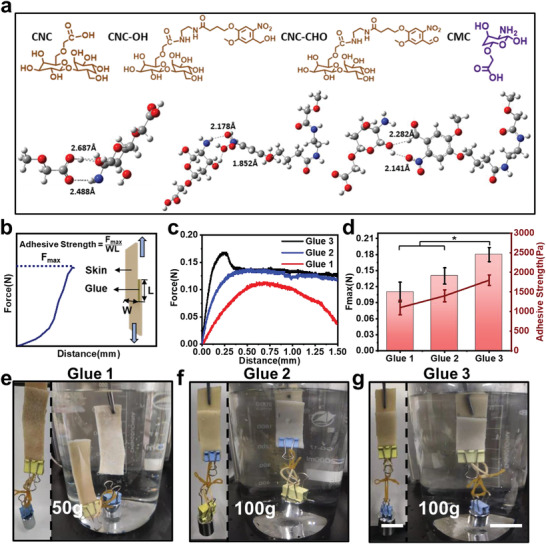
Simulation and adhesion capability of smart bio‐glues. a) Simulated interactions between CNC, CNC–OH, CNC—CHO, and CMC. b) Schematic representation of the pigskin adhesion measurement. c) Stress versus strain curves of bio‐glues on a pigskin. d) *F*_max_ and skin adhesive strength of the bio‐glues (*n* = 5). Optical images of wet adhesion properties of e) Glue 1, f) Glue 2, and g) Glue 3, respectively. (Scale bar: 25 mm) *p < 0.05, **p < 0.01, ***p < 0.001.

To further evaluate the adhesion capability of these smart bio‐glues under the irradiation of NIR light, the interactions between bio‐glues and tissues have been carefully explored. According to previous studies,^[^ ^43^, ^44^, ^46^ ^]^ CMC with amino groups could be selected as the mimics of human tissues, which also contribute to the bio‐glues construction. It was speculated that hydrogen bonding and electrostatic interactions may play vital roles in tissue adhesion besides the imine crosslinking between glues and tissues. To verify our hypothesis, density functional theory (DFT) was performed to simulate the non‐covalent interactions between different truncated molecules (Figure S7a, Supporting Information).^[^ ^47^, ^48^, ^49^ ^]^ As shown in **Figure** **2**a, the average bond length of the non‐covalent bonds between CNC, CNC‐OH, and CNC‐CHO with CMC were recorded as 2.59, 2.02, and 2.21 Å, respectively. Correspondingly, the binding energy was −81.58, −377.76, and −340.47 kcal mol^−1^, which confirmed the consistent trends. It was anticipated that the difference in the above outcomes may derive from the distinct charge density of those biopolymer chains. For CNC, only carboxyl groups could interact with the amines on CMC, while CNC–OH and CNC–CHO could interact with CMC via much stronger non‐covalent interactions owing to the existence of carboxyl, nitro, and amide groups. Therefore, these results confirm the significant role of the non‐covalent interactions between smart internal bio‐glues and tissue for bioadhesion.

The adhesion properties of bio‐glues were then quantitatively examined via a detailed adhesion measurement analysis (Figure 2b and Figure S7b, Supporting Information). As shown in Figure 2c, the *F*_max_ of the glues exhibited a UCNPs concentration‐dependent trend, which increased from 0.11 ± 0.02 to 0.18 ± 0.01 N from Glue 1 to Glue 3. Correspondingly, the adhesive strength of Glue 2 and Glue 3 were 1400 ± 152 and 1800 ± 132 Pa, respectively, which were higher than that of Glue 1 (1100 ± 186 Pa) (Figure 2d). These results showed that the tissue adhesion of Glue 3 after NIR light irradiation was stronger than that of Glue 1, which was because that UCNPs within the glues could efficiently convert NIR light into UV light as an internally secondary light source to induce the imine crosslinking reaction on tissue interface. Compared to previous tissue adhesives,^[^ ^22^, ^49^, ^50^, ^51^ ^]^ the bio‐glues developed in the current study could work well for the adhesion strength and wound healing in small animals (i.e., rats, rabbits) and even larger animals (i.e., pigs, dogs, monkeys). Finally, the wet adhesion properties of the glues to bind skin tissue were further investigated. Bio‐glues were applied onto a pig skin under UV and NIR light irradiation, and were then pressed gently (Figure S7c, Supporting Information). As shown in Figure 2e–g, the underwater testing results demonstrated that both Glue 2 and Glue 3 could adhere to the skin firmly in air and water, even lifting a 100 g weight, which proved the main adhesion interaction of Glue 3 after NIR irradiation was imine crosslinking. Conversely, Glue 1 could only approach 50 g in air, and less than 50 g in water, which might be due to some noncovalent interactions within Glue 1 destroyed in water. (Figure S7d, Supporting Information). The wet adhesion capabilities were also strengthened with the increase of UCNPs loading since higher concentration UCNPs could induce more imine crosslinking bond formation after being treated with the same NIR light irradiation time. Therefore, all aforesaid results demonstrated the outstanding adhesion capability of smart bio‐glues under NIR light irradiation for a wide range of internal wound healing and tissue bioadhesion applications.

### 2.3 Hemostatic Performance of Bio‐Glues

**Figure 3:**
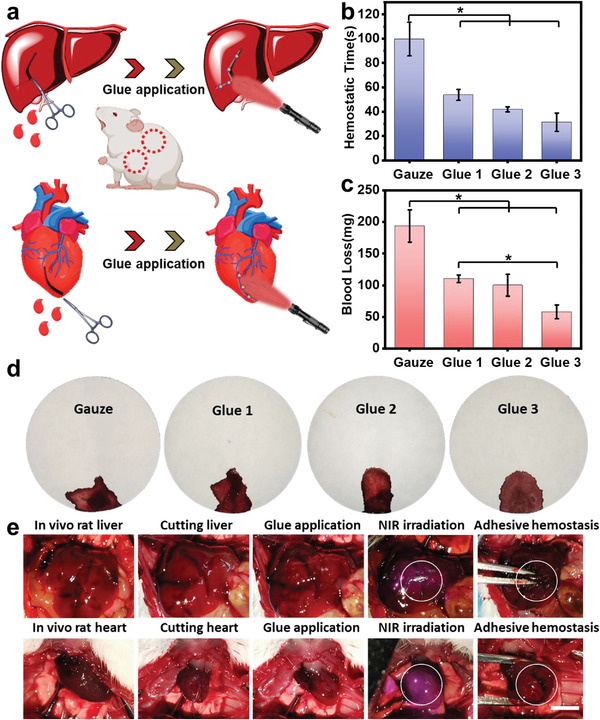
Tissue hemostatic properties of smart bio‐glues. a) Schematic illustration of the glues for rat liver and heart hemorrhage application. In vivo adhesion evaluation was performed by recording b) hemostatic time and c) blood loss (*n* = 4). d) Optical images of blood loss in wounds after different treatments. e) In vivo adhesion of Glue 3 on rat cutting liver and heart, respectively. The white circles represent the gel state. (Scale bar: 5 mm) *p < 0.05, **p < 0.01, ***p < 0.001.

To evaluate the hemostatic performance of as‐prepared glues, rat heart and liver injury models were established (**Figure** **3**a) by making a hole (diameter of 2 mm) in the liver via a syringe needle. The glues were then applied to the pinhole to inhibit fast bleeding, which was gently treated by UV and NIR light irradiation. The hemostatic time decreased remarkably by applying each bio‐glues (53.8 ± 4.53, 41.95 ± 2.05, and 31.35 ± 7.57 s, respectively) compared to the gauze control group (99.75 ± 13.79 s), and Glue 3 demonstrated the fastest hemostatic speed among the samples (Figure 3b). Meanwhile, we recorded the blood loss of glues in wet and dry situations, and the gauze group was found to perform the highest blood loss (193.71 ± 25.45 mg in wet and 45.75 ± 6.01 mg in dry) among all the groups. By applying three types of glue, blood loss significantly reduced to 110.35 ± 6.01, 99.95 ± 17.18, 57.85 ± 10.96 mg in wet and 20.9 ± 1.27, 20.05 ± 2.05, 16.2 ± 5.37 mg in dry, respectively (Figure 3c and Figure S8a, Supporting Information). As shown in Figure 3d, we intuitively observed a significant decrease in blood loss after being treated with Glue 3. Note that the hemostatic capabilities were also improved with the increase of UCNPs loading since higher concentration UCNPs could induce more imine crosslinking bond formation for tissue adhesion and hemostasis. To further investigate the bioadhesive performances on wet tissues in vivo, we continued to employ Glue 3 on rat liver and heart surface, respectively. Upon NIR irradiation for 120 s, Glue 3 instantly crosslinked and then firmly adhered to the surfaces of the wet tissues (Figure 3e). All these results showed that those smart bio‐glues possessed excellent hemostatic properties in vivo to fulfill the internal wound healing and tissue bioadhesion.

### 2.4 Biocompatibility and Tissue Bioadhesion of Bio‐Glues

**Figure 4:**
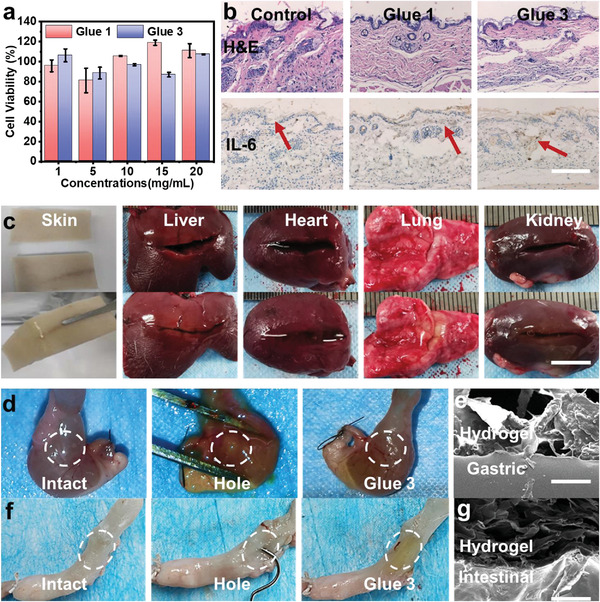
Biocompatibility and bioadhesion of smart bio‐glues. a) Cell viability of the glues with different concentrations (*n* = 5). b) H&E staining and IL‐6 immunohistochemical staining of the subcutaneous skin tissue after 7‐days treatment of rats. (Scale bar: 20 µm) c) Optical images of tissue adhesion in various cut damaged tissues. (Scale bar: 10 mm) d) Gastric perforation repair of Glue 3 and e) SEM image between Glue 3 after irradiation and gastric. (Scale bar: 100 µm) f) Intestinal perforation repair of Glue 3 after irradiation and g) SEM image between Glue 3 after irradiation and intestinal. (Scale bar: 100 µm)

To further assess the biocompatibility of those bio‐glues, the cell proliferation activity and the intracellular cytotoxicity in vitro were firstly evaluated by Alamar blue assay and Live/Dead staining assay. The NIH 3T3 cells incubated with the Glue 1 and Glue 3 after light irradiation extracts (20 mg mL^−1^) conditioned medium could similarly proliferate after 24 h culture in comparison to classical complete medium (Figure S8b, Supporting Information). Notably, the cell viability was still kept at ≈95% as the concentration increased, indicating the excellent biocompatibility of all glues (**Figure** **4**a). Subsequently, the biocompatibility and biodegradability of those bio‐glues in vivo were evaluated by histological staining assay of the collected specimens after implanting bio‐glues in the dorsal subcutaneous space of rats. Specifically, Glue 1 and Glue 3 were implanted into rat dorsal subcutaneous space, respectively, and then the skin tissues of implanted subcutaneous space were collected and fixed with 4% paraformaldehyde for histological analyses after 7 days. As shown in Figure 4b, hematoxylin‐eosin (H&E) staining demonstrated that the wounds treated with glues possessed similar epithelial tissues compared with the control group, which proved these smart glues did not affect the cells and tissue proliferation. Additionally, interleukin‐6 (IL‐6) immunohistochemistry analysis showed no more obvious positive expression in implanted tissue sites of all groups, which further demonstrated the low inflammatory stimulation and excellent biocompatibility. Note that bio‐glues did not appear at the subcutaneous space after 7 days, which indicated the good in vivo degradation of bio‐glues composed of biodegradable biopolymers (i.e., CMC, and CNC). Obviously, the degradation rate of bio‐glues can be tuned directly through the crosslinking density and the nature of different biopolymers. To achieve the optimized effect for wound healing, appropriate bio‐glues were needed for different wound and healing processes. In particular, for larger animal models, higher crosslinking density and more glues were usually needed for the bigger wound and slower healing process. More interestingly, these as‐prepared bio‐glues just induced a minimal inflammatory response after in vivo implantation, which is in line with previous reports.^[^ ^10^, ^12^, ^44^, ^46^ ^]^ All these results provided convenience and promise for wound healing and other adhesive applications in vivo.

To further study the tissue adhesive properties of bio‐glues, ex vivo adhesive performance was carried out in a wide range of tissues, including cutting and perforating tissues, destroyed gastric and intestinal. As shown in Figure 4c and Figure S8c, Supporting Information, 200 µL Glue 3 was injected onto different damaged tissues (including cut and perforate damaged, e.g., skin from pig; liver, heart, lung, and kidney from rat). After NIR light irradiation, the crosslinking network hydrogel was generated to fulfill tissues. Additionally, gastric and intestinal perforation models (perforation diameter of 2 mm) have been performed to evaluate the adhesive properties of bio‐glue for perforation repairing (Figure S8d, Supporting Information). The filling water in both gastric and intestines would flow out fast without any treatments while they possess the ability to refill the water after Glue 3 conglutinating with NIR irradiation. (Figure 4d,f). Subsequently, we observed the formation of tight micro‐interfaces between Glue 3 and tissues after bioadhesion (Figure 4e,g and Figure S8e, Supporting Information). Cross‐sectional SEM images showed that there was no obvious delamination between crosslinked Glue 3 and different tissue surfaces, which was due to the formation of imine crosslink and multiple non‐covalent bonds at the interface. It was further observed that Glue 3 remained the porous structure after irradiation, which promoted blood adsorption and internal wound healing. Above all, these results indicated that bio‐glues with excellent biocompatibility and adhesion performances could be used for a variety of internal tissue bioadhesion and wound conglutination.

### 2.5 Bio‐Glues for External Wound Healing

**Figure 5:**
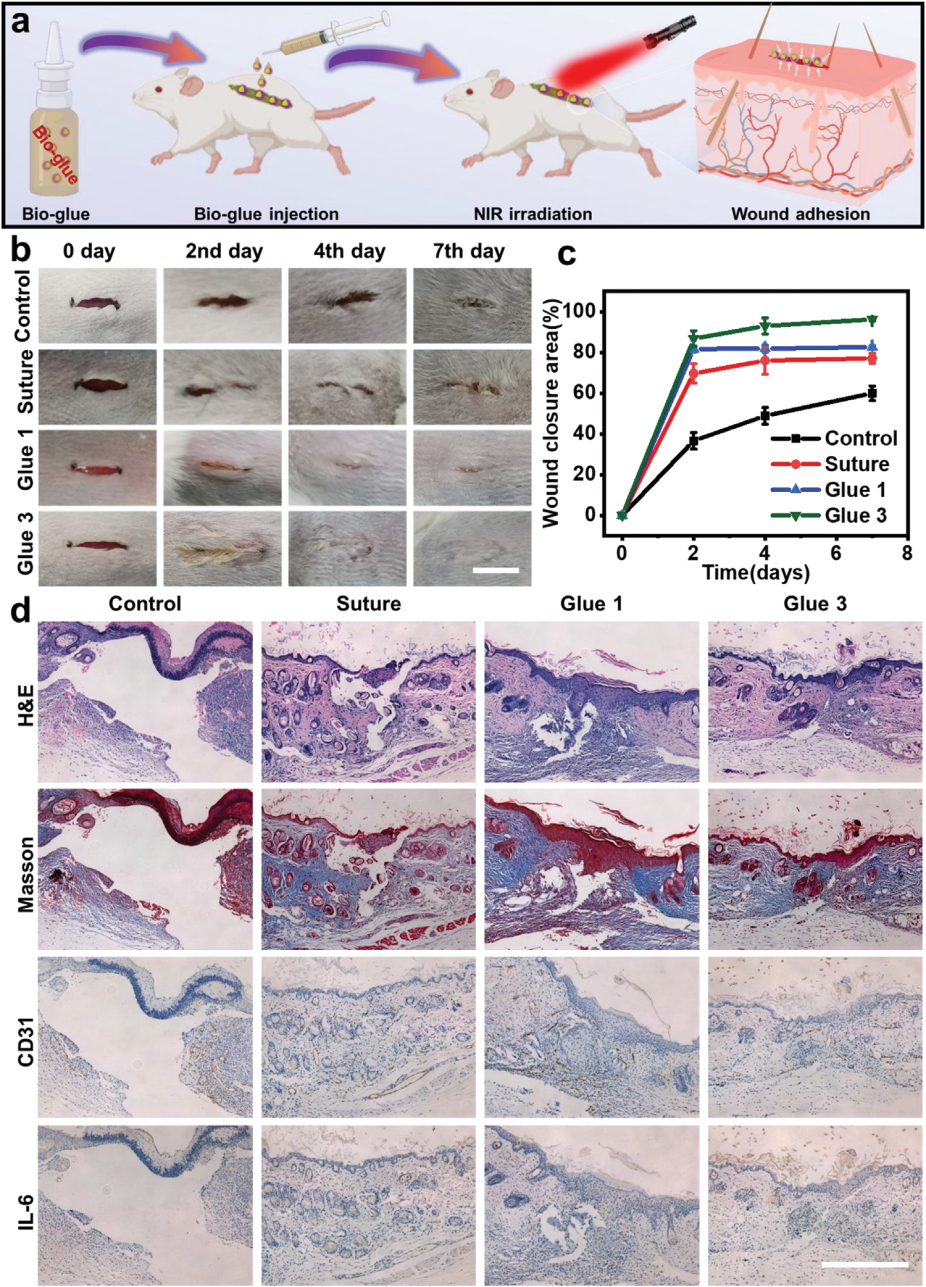
Smart bio‐glues for external wound healing in a rat model. a) Schematic representation of bio‐glues application in vivo for linear wound healing. b) Representative photographs of wound recovery at 0, 2nd, 4th, and 7th day in all groups. (Scale bar: 10 mm) c) Corresponding wound closure area statistics of different treatments. d) Representative histological H&E staining, Masson's trichrome, immunohistochemical CD31, and IL‐6 staining of the skin tissues after 7 days. (Scale bar: 20 µm)

We next performed the systematic in vivo bio‐evaluations of those bio‐glues in wound healing on both external and internal wound systems using rat and rabbit models. For external wound conglutination, rat skin customized linear incisions were investigated (**Figure** **5**a). As shown in Figure 5b, animal wounds treated with surgical sutures, Glue 1, and Glue 3 groups were all able to improve the wound conglutinating, and the glues‐assisted groups exhibited better performances compared with the surgical suture and untreated control group at second, fourth, and seventh day. After each treatment, the reduced wound area of surgical sutures, Glue 1, and Glue 3 groups reached ≈70%, ≈80%, and ≈85% on the 2nd day, respectively, suggesting that these bio‐glues could perform better tissue conglutination and confirmed fungibility compared to traditional suture (Figure 5c). Moreover, the linear wound treated with Glue 3 closed entirely on the seventh day, while conspicuous wounds remained in the surgical suture and untreated control group (Figure S9, Supporting Information), demonstrating the excellent capacity of bio‐glue for regenerating skin.

To further verify the recovery of the healed skin tissue, histological analysis including H&E staining, Masson's trichrome staining, platelet endothelial cell adhesion molecule‐1 (CD31), and IL‐6 immunohistochemical staining were carefully performed (Figure 5d). The H&E and Masson's staining revealed that there are more complete epithelial tissues and more collagen fibers in the glue‐treated groups, while the neo‐epidermis and epithelial tissues of the wounds in control and surgical suture groups were still rough and irregular on the seventh day (Figure S10a, Supporting Information). On the other hand, the wound treated with Glue 3 contained the most complete epithelial tissues, which demonstrated that the smart bio‐glue could indeed accelerate wound healing. In addition, it was reported that platelet endothelial cell adhesion molecule‐1 (CD31) immunohistochemical staining could play a key role in endothelial cell growth and angiogenesis.^[^ ^52^ ^]^ And the group treated with glues showed stronger expression, which indicated the glues could induce better endothelial cell growth and angiogenesis capability compared to other groups. Moreover, we further evaluated the wound inflammation by IL‐6 immunohistochemical analysis. After 7 days of treatment, the lowest level of IL‐6 was detected in the glue‐treated groups, and the relative level of inflammatory responses of untreated control, surgical sutures, Glue 1, and Glue 3 groups reached ≈0.2, ≈0.17, ≈0.14, and ≈0.10 after 7 days, respectively, indicating the good tissue biocompatibility of bio‐glues. (Figure S10b, Supporting Information). Overall, these results supported the smart internal bio‐glues demonstrated excellent tissue adhesion and hemostatic performances in external wound healing, which provided the alternative for surgical sutures in clinical research.

### 2.6 Bio‐Glues for Internal Wound Healing

**Figure 6:**
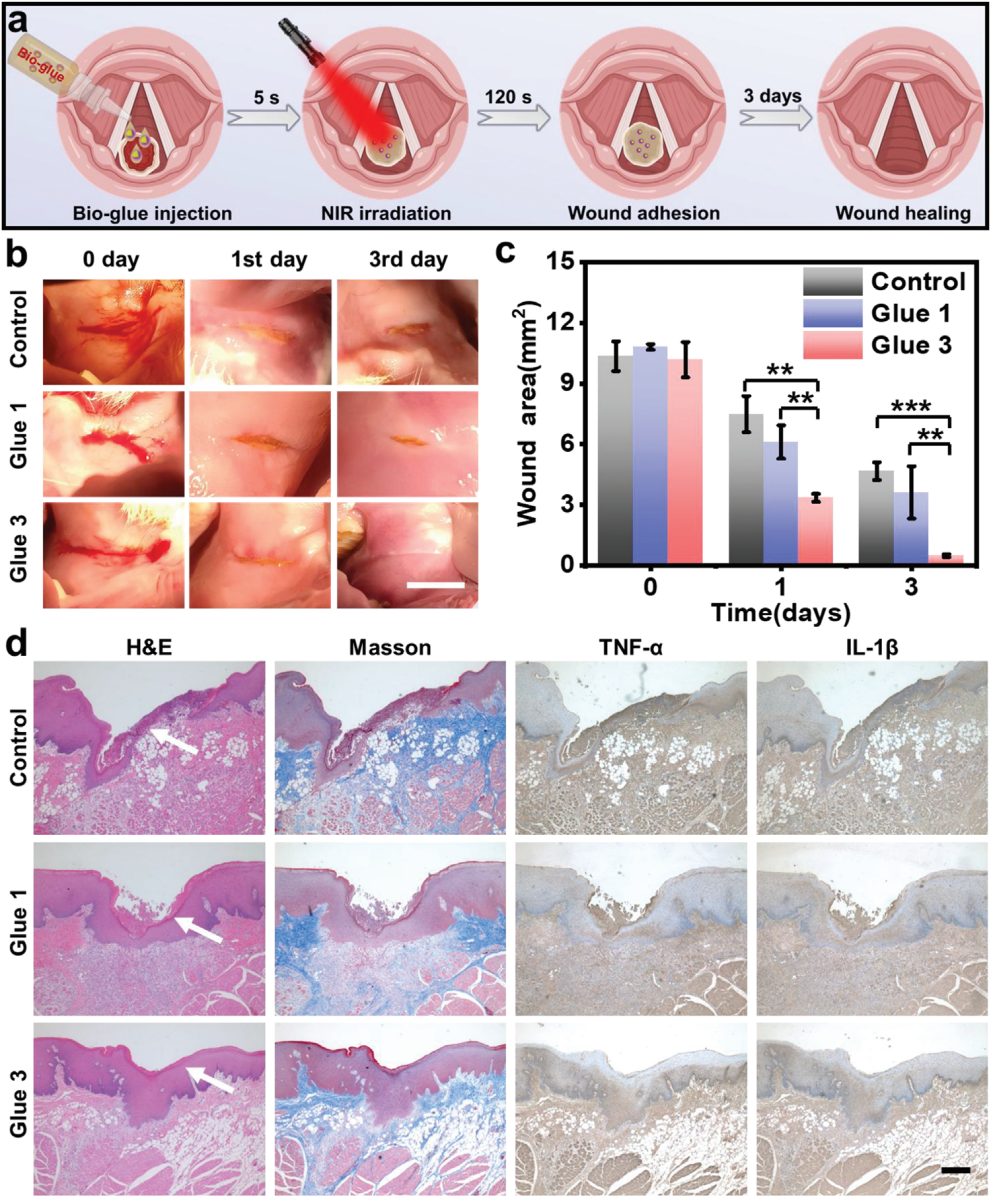
Smart internal bio‐glues for internal wound healing in a rabbit model. a) Schematic illustration of bio‐glues application in vivo for throat wound healing. b) Representative photographs of wound area at 0, 1st, and 3rd day in all groups. (Scale bar: 10 mm) c) Statistics analyses of wound area after different treatments (*n* = 4). d) Representative images of histological H&E staining, Masson's trichrome staining, TNF‐*α*, and IL‐1*β* immunohistochemical staining of the skin tissues after 3‐days treatment (Scale bar: 20 µm). *p < 0.05, **p < 0.01, ***p < 0.001.

To further investigate the potential application and underlying mechanism of bio‐glues for internal wound conglutination, the bio‐glues were further applied for the rabbit throat wound model treatment (**Figure** **6**a and Figure S11, Supporting Information). In brief, rabbit throat tissue wounds with a 10 mm linear incision were prepared. Then, Glue 1 and Glue 3 were dropped onto the wound through the catheter while the light irradiation was performed simultaneously outside the neck skin (Figure S12a and Movies S1 and S2, Supporting Information). In this case, Glue 1 was irradiated with UV, which served as a control for the comparison of in situ UV and NIR light effects on the gelation and adhesion properties of glues. As shown in Figure 6b, the linear incision exhibited no obvious acceleration after Glue 1 treatment, while rapid conglutination and healing could be observed in Glue 3 group. Besides, the marks of the incision showed a fast disappearance than Glue 1 and the control group on the third day. The wound area after each group treatment was also calculated in Figure 6c, and the wound area of untreated control, Glue 1, and Glue 3 groups reached 4.66, 3.62, and 0.29 mm^2^ on the third day, respectively. This result suggested that the smart bio‐glues could perform high internal wound healing and regeneration efficiency via portable operation‐induced conglutination.

Moreover, histopathological observations including H&E staining, Masson's trichrome, tumor necrosis factor‐*α* (TNF‐*α*), and interleukin‐1*β* (IL‐1*β*) immunohistochemical staining were also performed to evaluate the internal wound healing effect. (Figure S12b, Supporting Information). As shown in Figure 6d, H&E staining revealed minimal injury and more complete epithelial tissue in Glue 3‐treated group, while the tissues of the wounds in the control group and the Glue 1‐treated group were still irregular on the third day (Figure S13a, Supporting Information). In addition, Masson's trichrome staining revealed that more ordered collagen fiber formation around the wound healing area could be observed in Glue 3‐treated group. Notably, the pro‐inflammatory cytokines including TNF‐*α* and IL‐1*β* immunohistochemical staining showed that the lower level could be detected in Glue 3‐treated group, and the relative level of inflammatory responses in untreated control, Glue 1, and Glue 3 groups reached ≈0.35, ≈0.3, and ≈0.25 on the third day, respectively, which indicated its lower inflammation and good biocompatibility (Figure S13b,c, Supporting Information). Therefore, these results confirmed that the smart internal bio‐glues would be a benefit for internal wound healing via portable NIR triggering, outstanding tissue adhesion, ordered collagen fiber formation, and low inflammatory stimulation, which demonstrated that the smart bio‐glue could be a promising candidate for internal wound conglutination and healing. We also believed that the as‐prepared bio‐glues could work well in internal wound repair in larger animals (i.e., pigs, dogs, and monkeys) by carefully adjusting the parameters including UCNP concentrations, NIR power, and irradiation time, which can provide new opportunities for minimally invasive surgery, especially in internal wound healing.

## 3 Conclusion

In summary, smart internal bio‐glues have been successfully fabricated via the combined usage of NB‐modified biopolymers and UCNPs. Those bio‐glues possessed outstanding adhesion, excellent hemostatic performances, and biocompatibility, which were applied for tissue adhesion and hemostasis with the pigskin‐covered under NIR light irradiation. Besides, it was found that the presence of UCNPs would not influence the formation of imine‐crosslinking and non‐covalent bonds between glues and tissues. Moreover, the NIR light‐responsive bio‐glues with deeper tissue penetration ability were conducive to various tissue adhesion and wound healing, which has been confirmed by the fast wound healing testing such as the rat skin wound healing (within 7 days) and rabbits’ internal wound healing (within 3 days). We believed that this work could provide new opportunities for minimally invasive surgery, especially in internal wound healing using smart and robust bio‐glues.

## 4 Experimental Section

### Materials

4‐hydroxy‐3‐methoxybenzaldehyde (Vaniline, 99%), methyl 4‐bromobutyrate (98%), p‐Toluenesulfonic acid monohydrate (99%), YbCl_3_ (99%), TmCl_3_ (99%), and oleic acid (90%) were purchased from Adamas‐beta (Shanghai, China). Sodium borohydride (NaBH_4_, 97%) was purchased from Kermel Chemical Reagent Co., Ltd. (Tianjin, China). Carboxymethyl cellulose (CNC, M.W. 250 000, degree of substitution 90%), polyacrylic acid (PAA), gelatin (Gel), hyaluronic acid (HA), and collagen polypeptide (COL) were purchased from Aladdin Biochemical Technology Co. Ltd (Shanghai, China). Carboxymethyl chitosan (CMC, M.W.100 000–300 000, degree of substitution 95%) was purchased from Santa Cruz Biotechnology (California, USA). 3‐(3‐dimethylaminopropyl)‐1‐Ethylcarbodiimide hydrochloride (EDC·HCl, 97%) was purchased from Titan Scientific Co. Ltd (Shanghai, China). N‐Hydroxysuccinimide (NHS, 98%), and 1‐Hydroxybenzotrizole (HOBt, 98%) were purchased from J&K Scientific Ltd. (Beijing, China). YCl_3_ (99%), NH_4_F (98%), and 1‐octadecene (90%) were purchased from Alfa Aesar (Shanghai, China). Sodium alginate (SA, 99%), ethylenediamine (99%), cyclohexane (99%), ethanol (99%), methyl alcohol (99%), ethyl acetate (99%), petroleum ether (99%), acetone (99%), NaOH (99%), K_2_CO_3_ (99%), MgSO_4_ (99%), HNO_3_ (99%), HCl (99%), DCM (99%), THF (99%), and DMF (99%) were acquired from Kelong Chemical Reagent Co., Ltd. (Chengdu, China). All chemical reagents were freshly used without any further purification in the research.

### Characterization

The surface morphologies of the cross‐section were acquired on SEM of FEI Quanta 250. The transmission electron microscope (TEM) images were acquired on Talos F200i (Thermo Scientific, Czech). ^1^H NMR spectroscopy was performed on AV III HD 400 MHz of Bruker. The ESI‐MS spectrum was applied on Applied Biosystems API 2000 with negative anion mode electrospray ionization with the 10 µL min^−1^ flow rate. UV–vis spectroscopy was performed by PerkinElmer Lambda 650 UV–vis spectrophotometer. Fourier‐transform infrared spectroscopy spectra were conducted with PerkinElmer spectrum one B system using KBr pellets methods with the resolution of 4.0 cm^−1^. XPS measurements were performed with a VG ESCALAB MKII spectrometer. XPSPEAK software (version 4.1) was used to deconvolute the narrow‐scan XPS spectra of C ^1s^, O ^1s^, N ^1s^, F^1s^, Y^3d^, Na^1s^, Yb^4d^, and Tm^4d^ of the sample, and performed baseline calibration before use.^[^ ^24^, ^53^, ^54^ ^]^ The injectability of the glue was carried out with needles. The viscoelastic properties of the hydrogel samples were monitored by a rheological measurement (MCR320, Austria). The diameter of the parallel plate is 8 mm, and the experimental temperature is constant at 25 °C. Time sweep tests were performed with 1% constant strain and 10 rad s^−1^ constant frequency. Frequency sweep tests were performed from 100 to 0.1 rad s^−1^ with 1% constant strain. The swelling ratio (%) of the hydrogel was calculated by dividing the real‐time mass *W*_1_ by the initial mass *W*_0_ under complete immersion in water at different times. The tensile testing machine (INSTRON, USA) was used to measure adhesion abilities.

### Synthesis and Characterization of NB and Biopolymer‐NB

Methyl4‐(4‐(hydroxymethyl)‐2‐methoxy‐5‐nitrophenoxy) butanoate (mNB) was synthesized according to the previous article.^[^ ^21^ ^]^ Subsequently, the photosensitive structure o‐nitrobenzyl alcohol (NB) was synthesized from mNB.^[^ ^43^ ^]^ The NB chemical structure was fully confirmed by the ^1^H nuclear magnetic resonance (NMR) spectrum, electrospray ionization mass spectrometry (ESI‐MS) spectrum, UV–vis spectrum, and Fourier transform infrared (FTIR) spectrum.

Then, NB was successfully grafted onto the side chain of biopolymers (i.e., polyacrylic acid (PAA), gelatin (Gel), sodium alginate (SA), carboxymethyl chitosan (CMC), carboxymethylcellulose (CNC), hyaluronic acid (HA), collagen polypeptide (COL)) through amidation reaction with different carboxyl activator.^[^ ^43^, ^44^ ^]^ In short, to avoid biopolymer crosslinking, 0.2 g of biopolymers were dissolved in 40 mL of deionized water at 37 °C and stirred for 2 h. Then 250 mg EDC·HCl was added to the above solution and stirred for 30 min, and subsequently, 10 mg NB and 150 mg HOBt (HA) or NHS (others) were dissolved in 4 mL deionized water and added dropwise to the mixed solution. The pH was adjusted to about 5 with 1M HCl. Finally, the above solution was continuously stirred for 48 h. The obtained solution was put into a dialysis bag (MWCO: 14000, Spectrum), and dialyzed with deionized water for 5 days. The mixture was lyophilized to obtain biopolymer‐NB. ^1^H NMR spectroscopy, UV−vis spectrophotometry, and FTIR spectroscopy were used to prove the successful grafting of biopolymer‐NB.

### Synthesis of UCNPs

Lanthanide‐doped up‐conversion nanoparticles (*β*‐NaYF_4_: 30% Yb^3+^ / 0.3% Tm^3+^ UCNPs) with core‐shell structures containing oleic acid ligands were successfully synthesized according to previous literature.^[^ ^42^ ^]^ To make UCNPs dispersed in the bio‐glues, further processing to obtain aqueous UCNPs: firstly, the UCNPs dispersed in cyclohexane were centrifuged to obtain solid components, and then dispersed in 0.1 M HCl. The product was sonicated at 45 °C for over 1 h and collected by centrifugation, as well as re‐dispersed in acetone and then collected by centrifugation again. Finally, UCNPs were dispersed in the aqueous solution with 10 mg mL^−1^ and sonicated for 5 min prior to use.

### Preparation of Bio‐Glues

UCNPs were dispersed in deionized water with different concentrations, with mass volume percentages(w/v) of 0%, 0.5%, and 1%. Then CNC‐NB and CMC were added to the above solution with the concentration of 60 mg mL^−1^, and the detailed ratios were shown in Table S1, Supporting Information. The CNC‐NB/CMC hydrogel precursor solutions with 0%, 0.5%, and 1% (w/v) UCNPs were named as Glue *i* (*i*  =  1 to 3), respectively.

### Preparation of Hydrogels With and Without Skin

To verify and compare the penetration depth of UV and NIR on the skin tissue, the formation of hydrogels with and without a pigskin‐covered was evaluated. Firstly, 365 nm UV light (power: 2 W) was selected as the control, and 980 nm NIR light (power: 2 W) was selected for the preparation and study of bio‐glues. Then, the glues were injected into a cylindrical silicone mold with a height of 1 mm and a diameter of 10 mm. Next, Glue 1 was irradiated with 365 nm UV light and Glue 2 and Glue 3 were irradiated with 980 nm NIR light without skin at a distance of about 1.5 cm for 120 s to form CNC‐NB/CMC/UCNPs hydrogels. Finally, glues were covered by tiny pigskins (50 × 25 × 3 mm) and irradiated with UV and NIR light as described above for the preparation of hydrogels.

### Computer Theoretical Calculation

Density functional theory (DFT) calculations were used to study the electrostatic interactions between different truncated molecules (CNC, CNC‐OH, CNC‐CHO) with CMC, and explain the adhesion mechanism.^[^ ^47^, ^48^, ^49^ ^]^ The geometry optimizations were carried out at the B3LYP/6‐31 g (d, p) level. Based on the optimized structure, the vibration frequency analysis of all molecules was carried out at the same time. Gibbs's free energies were calculated from harmonic frequencies under 298.15 K after the stability of the optimized structures was also confirmed.

### Adhesion Capability Testing

The universal material tensile testing machine (INSTRON, USA) was used to measure the pigskin adhesion force for further tissue adhesion strength analyses of the glues. In short, a lot of pigskins (25 × 10 × 3 mm) were prepared, then 100 µL of Glue *i* (*i* = 1 to 3) was evenly coated on the surface of a 10 × 10 mm area. Then Glue 1 was irradiated with a 365 nm UV light at a distance of ≈1.5 cm for 120 s to produce adhesion. Glue 2 and Glue 3 were irradiated with 980 nm NIR light at a distance of ≈1.5 cm for 120 s. Finally, the samples (*n* = 5) were loaded on the machine and stretched at a tensile speed of 0.01 mm s^−1^ until broken. The recorded maximum tensile force (*F*_max_) was divided by the adhesion area to calculate the tissue adhesion strength.

### Adhesion Strength Testing in the Air and Underwater

A lot of pigskins (50 × 25 × 3 mm) were prepared. Then 200 µL Glue *i* (*i* = 1 to 3) was evenly coated on the surface of 25 × 25 mm. Then Glue 1 was irradiated with a 365 nm UV light at a distance of ≈1.5 cm for 120 s. Glue 2 and Glue 3 were irradiated with a 980 nm NIR light at a distance of ≈1.5 cm for 120 s to produce the adhesion. Then, the adhesive strength of Glue *i* (*i* = 1 to 3) was recorded and compared by a weight tensile test in the air and underwater.

### Hemostatic Testing of Bio‐Glues

All rats’ procedures were approved by the Animal Care and Experiment Committee of West China Hospital of Stomatology affiliated with the School of Medicine, Sichuan University (WCHSIRB‐D‐2017‐263). According to the previous studies,^[^ ^44^, ^55^, ^56^ ^]^ the bleeding models were established by partly cutting the heart and liver with scissors, and the prepared bio‐glues were injected onto the wound area immediately. In brief, twelve rats (25 ± 5 g) were randomly divided into four groups (*n* = 4), and then their liver lobes of them were applied as models. A 1 mL syringe was used to make a small hole in the liver, then the glue was quickly injected into the hole, and subsequently, the light was applied. During the operation, the blood loss was collected with filter papers, and the time to stop bleeding was recorded. Medical gauze and Glue 1 were used as the control, and the evaluation criterion was determined by weighing the paper in the wet and dry states. Next, the heart and liver were cut in vivo by a surgical scissor. Then, Glue 3 was immediately injected onto the defect sites. Upon NIR irradiation, hydrogel crosslinking adhesion was formed and the bleeding was stopped.

### In Vitro Biocompatibility and Cell Proliferation

The cell viability in vitro of each sample was evaluated by Alamar blue assay. Briefly, Glue 3 and Glue 1 after light irradiation were soaked in the culture medium for 24 h to obtain a 40 mg mL^−1^ leaching solution. Then, NIH 3T3 cells were incubated in a 96‐well plate at a density of 2000 cells per well for 24 h. Then, after being treated with leaching solution in different concentrations (1, 5, 10, 15, and 20 mg mL^−1^) for another 24 h, the Alamar blue assay was conducted to evaluate the corresponding cell viability (n = 5). Additionally, NIH 3T3 cells were incubated in a 6‐well plate at a density of 100 000 cells per well for 24 h. Then, after treating with the leaching solution (20 mg mL^−1^) for another 24 h, the Live/Dead staining was recorded using an inverted fluorescence microscope to confirm the NIH 3T3 cell viability and morphology.

### In Vivo Biocompatibility

Fifteen rats (25 ± 5 g) were randomly divided into three groups. To assess the biological toxicity of Glue 1 and Glue 3 in vivo, 50 µL of glues were injected into the back of the experimental groups. Untreated blank mice used saline as the control. After feeding for seven days, the injection site was removed from the animals for histopathological examination, including hematoxylin‐eosin (H&E) and interleukin‐6 (IL‐6) staining.

### Tissue Adhesion of Bio‐Glues

The liver, kidney, lung, and heart were obtained from rats (200 ± 5 g) and pigskins were purchased from the local market. For all the organs, the incision about 2 cm in length was made with a scalpel blade, and 200 µL Glue 3 was injected into the wound. Under the 980 nm laser irradiation, the incision of all organs from rats could have adhered. Additionally, for two separated pigskins, the two tissue pieces were glued together according to the same treatment.

### Gastric and Intestinal Perforation Repair

Firstly, the intact gastric and intestine of rats (200 ± 5 g) were filled with water, and water in the holes (perforation diameter of 2 mm) quickly flew out. Next,100 µL Glue 3 was injected into the holes, and irradiated with 980 nm light. It was observed that the holes were glued together and the gastric and intestine could be filled with water again.

### External Wound Healing of Rats

To evaluate the external wound healing application of glues, a wound healing model was established and conducted on a rat skin with a length of 10 mm as an alternative to surgical sutures. In short, the back skins of rats (25 ± 5 g) were shaved and disinfected, and the wound was prepared with a scalpel. The rats were randomly divided into 4 groups (surgical sutures, Glue 1, Glue 3, and control; surgical sutures and Glue 1 were used as experimental control; *n* = 4). Then, 50 µL Glue 1 and Glue 3 were dropped onto the wound, and the light was used to irradiate the wound. The suture treatment was also used, and the untreated rats were used as the control group. Then the rats were fed normally for 7 days, and the wound area was photographed and recorded. The wound closure area for rats in each group was estimated as follows:

*W* = (*W*_0_−*W*_i_)/*W*_0_ × 100%, where *W*_0_ was the initial wound area, *W*_i_ was the remained area of the wound on the 0th, 2nd, 4th, and 7th day accordingly. All animals were sacrificed after 7 days, and the skin tissues were obtained to perform the histopathological examination, including H&E staining, Masson's trichrome, platelet endothelial cell adhesion molecule‐1 (CD31), and IL‐6 staining.

### Internal Wound Healing of Rabbits

All rabbit experiments were approved by the Animal Ethics Committee of West China Hospital, Sichuan University (K2021031). To further explore the potential difference of bio‐glues in vivo, rabbit internal wound healing was performed on throat tissues with a 10 mm linear wound. In short, the rabbits (2 ± 0.05 kg) were randomly divided into 3 groups (Glue 1, Glue 3, and control; Glue 1 was used as experimental control; two rats per group). Then, two wounds were prepared on both sides of each rabbit's throat skin with a surgical knife. Then, 100 µL Glue 1 and Glue 3 were dropped into the wound, and the light was used to irradiate the wound outside the neck skin. The untreated blank defect was used as the control group. Then the rabbits were fed normally for three days, and the wound area was photographed and recorded every day. The wound area in each group was recorded on the 0th, 1st, and 3rd day. All animals were sacrificed after complete closure, and the throat tissues were obtained to perform the histopathological examination, including H&E staining, Masson's trichrome staining, tumor necrosis factor‐*α* (TNF‐*α*), and interleukin‐1*β* (IL‐1*β*) staining.

### Histomorphological Determination

All animals were sacrificed after complete closure, and samples were collected for biochemical analysis and histomorphological determination (*n* = 5). For evaluation of wound healing area, the wound distance was evaluated by estimating the wound location in H&E staining using a hematoxylin and eosin staining kit. (Shanghai Biyuntian Biological Co., Ltd., China). For evaluation of inflammation in the wound area, the samples were collected and then stained with tumor necrosis factor‐*α* (TNF‐*α*), interleukin‐6 (IL‐6), and interleukin‐1*β* (IL‐1*β*) staining kit. (Shanghai Biyuntian Biological Co., Ltd., China). And the fluorescent optical density and area were analyzed by ImageJ (Java, America), and the relative level was calculated by dividing the optical density by the area.

### Statistical Methods

SPSS software (version 20.0) was used for data analysis. The results were described as the mean ± standard deviation (SD) and quantified for each experimental group. One‐way analysis of variance was used for comparisons between the two groups, and *p* < 0.05 was considered to be statistical significance.

## Conflict of Interest

The authors declare no conflict of interest.

## Supporting information

Supporting Information

Supplemental Movie 1

Supplemental Movie 2

## Data Availability

The data that support the findings of this study are available in the supplementary material of this article.
