# Supplementary material for: Smart Internal Bio‐Glues
Source: Adv Sci (Weinh). 2022 Jul 28;9(27):2203587. doi: 10.1002/advs.202203587 (PMC9507370; doi:10.1002/advs.202203587)
Supplement: Supplementary file 1 — Supporting Information [file ADVS-9-2203587-s003.pdf]

ADVANCED SCIENCE
Open Access

Supporting Information

for *Adv. Sci.*, DOI 10.1002/advs.202203587

Smart Internal Bio-Glues

Hengjie Zhang, Jianhua Zhang, Xu Peng, Zhan Li, Wanjie Bai, Tianyou Wang, Zhipeng Gu and Yiwen Li**

Supporting Information

Smart Internal Bio-glues

Hengjie Zhang[1], Jianhua Zhang[1] , Xu Peng[2], Zhan Li[1], Wanjie Bai[1], Tianyou Wang[1], Zhipeng Gu[1,], Yiwen Li[1,]**

[1]College of Polymer Science and Engineering, State Key Laboratory of Polymer Materials Engineering, Sichuan University, Chengdu 610065, China.
[2]Experimental and Research Animal Institute, Sichuan University, Chengdu 610041, China.

E-mail: ywli@scu.edu.cn (Y.L.), guzhipeng2019@scu.edu.cn (Z.G.) Tel: +86 028-85401066

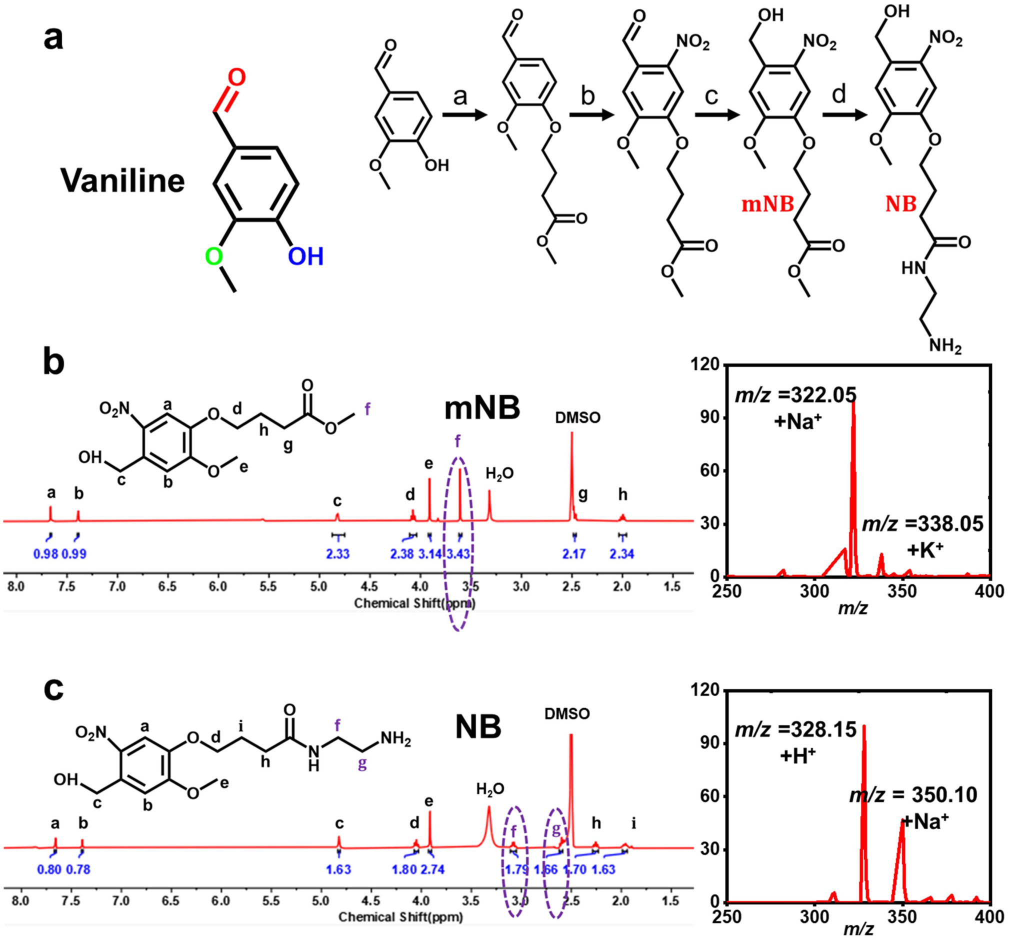

Figure S1. Synthesis and characterization of mNB and NB. (a) Synthetic route towards mNB and NB. ^{1}H NMR spectra and ESI-MS spectra of (b) mNB and (c) NB.

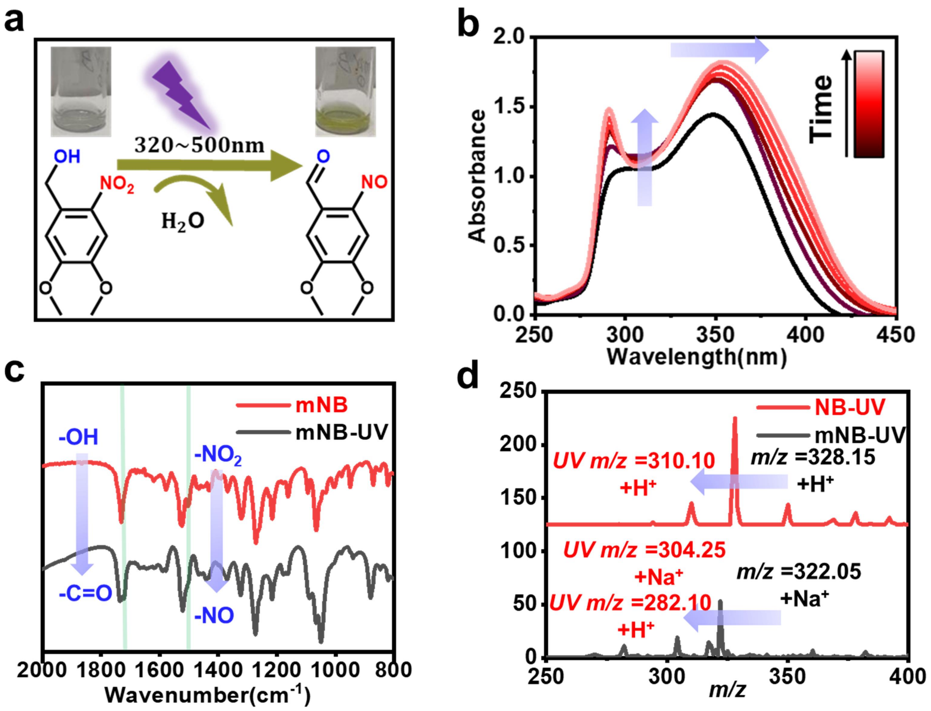

Figure S2. Characterization of UV light-responsive behavior of mNB and NB. (a) Structure and optical images of mNB and NB. (b) UV-Vis spectra of mNB with increasing irradiation time. (c) FTIR spectra of mNB as well as mNB irradiated for 5 min. (d) ESI-MS spectra of mNB and NB irradiated for 5 min. The arrow refers to the direction of UV irradiation.

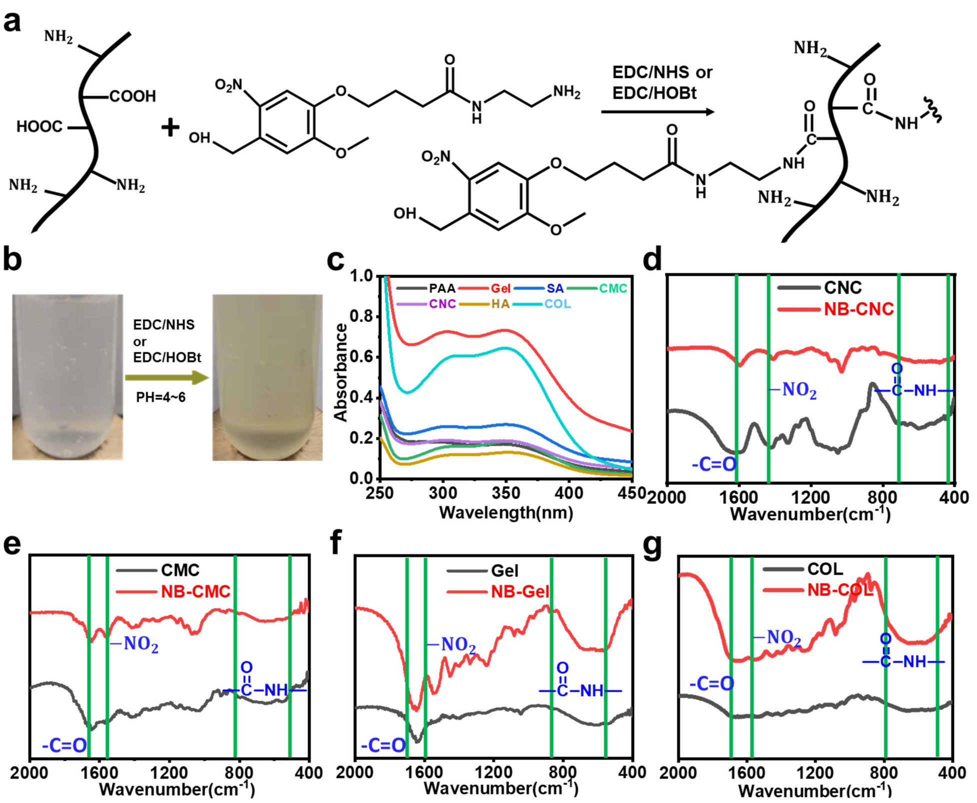

Figure S3. Synthesis and characterization of NB-biopolymers. (a)Synthetic route toward NB-biopolymers (HA: EDC/HOBt, Others: EDC/NHS). (b) Optical images of CNC solution and NB-CNC solutions. (c) UV-Vis spectra of NB-biopolymers. (d)~(g) FTIR spectra of NB-CNC, NB-CMC, NB-Gel, NB-COL.

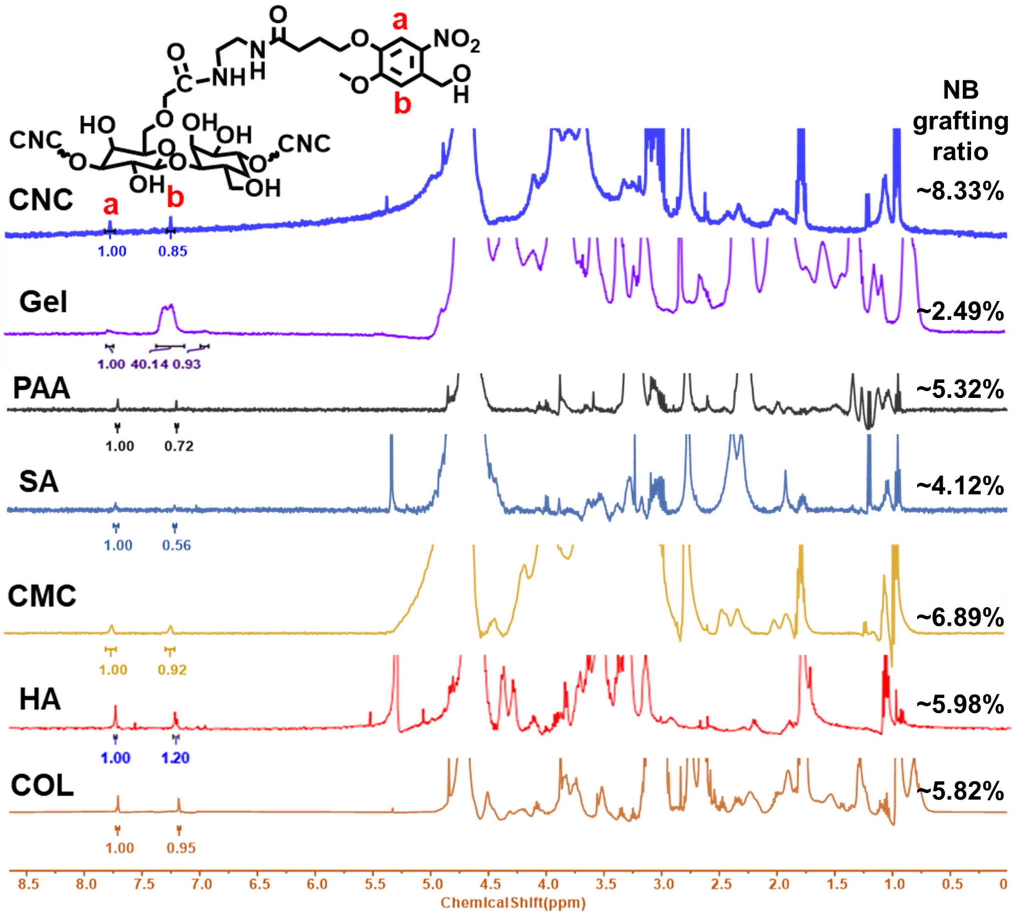

Figure S4. Chemical Structure of NB-CNC and ^{1}H NMR spectra of NB-biopolymers (HA: EDC/HOBt, Others: EDC/NHS).

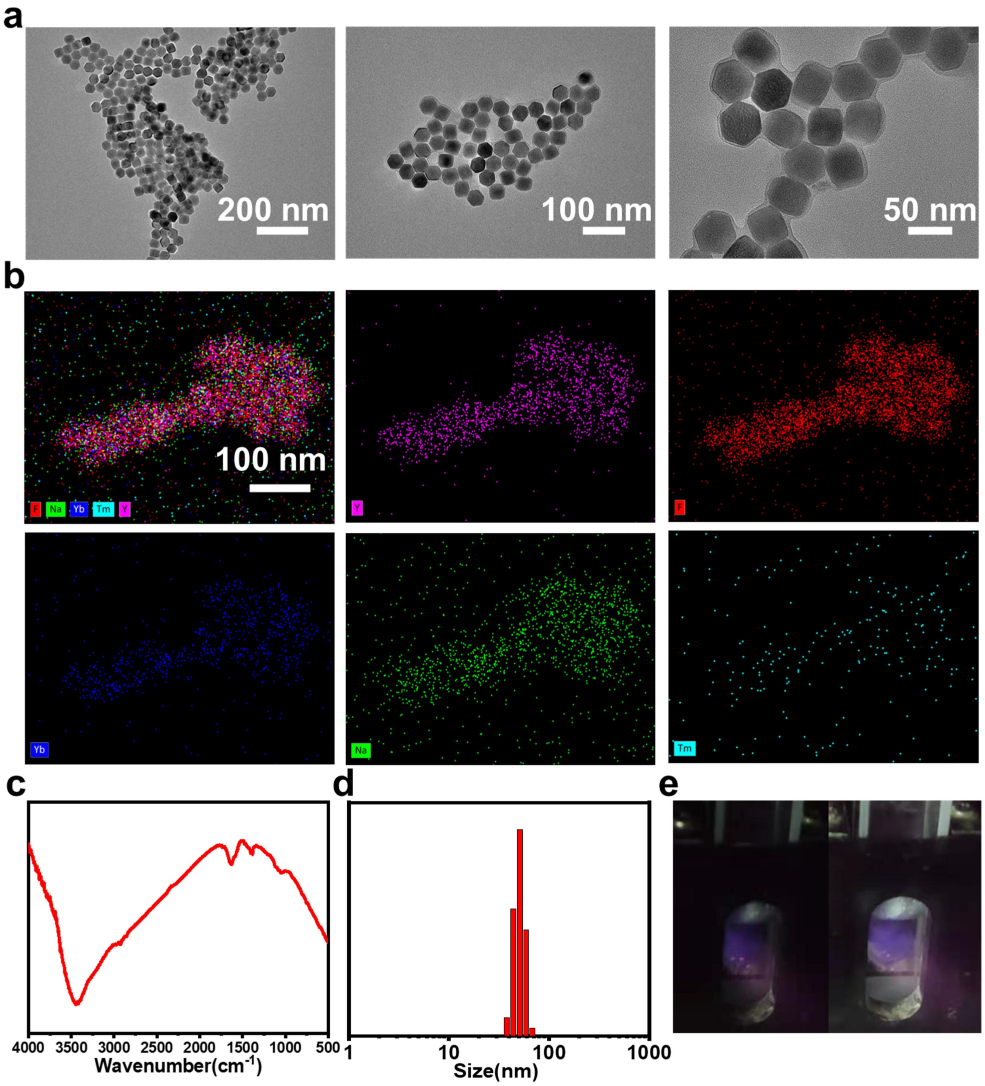

Figure S5. Synthesis and characterization of UCNPs. (a) TEM images, (b) element mapping images, and (c) FTIR spectra of UCNPs. (d) Hydrodynamic size of UCNPs determined by DLS. (e) Optical images of UCNP irradiated by NIR light with daylight or under dark.

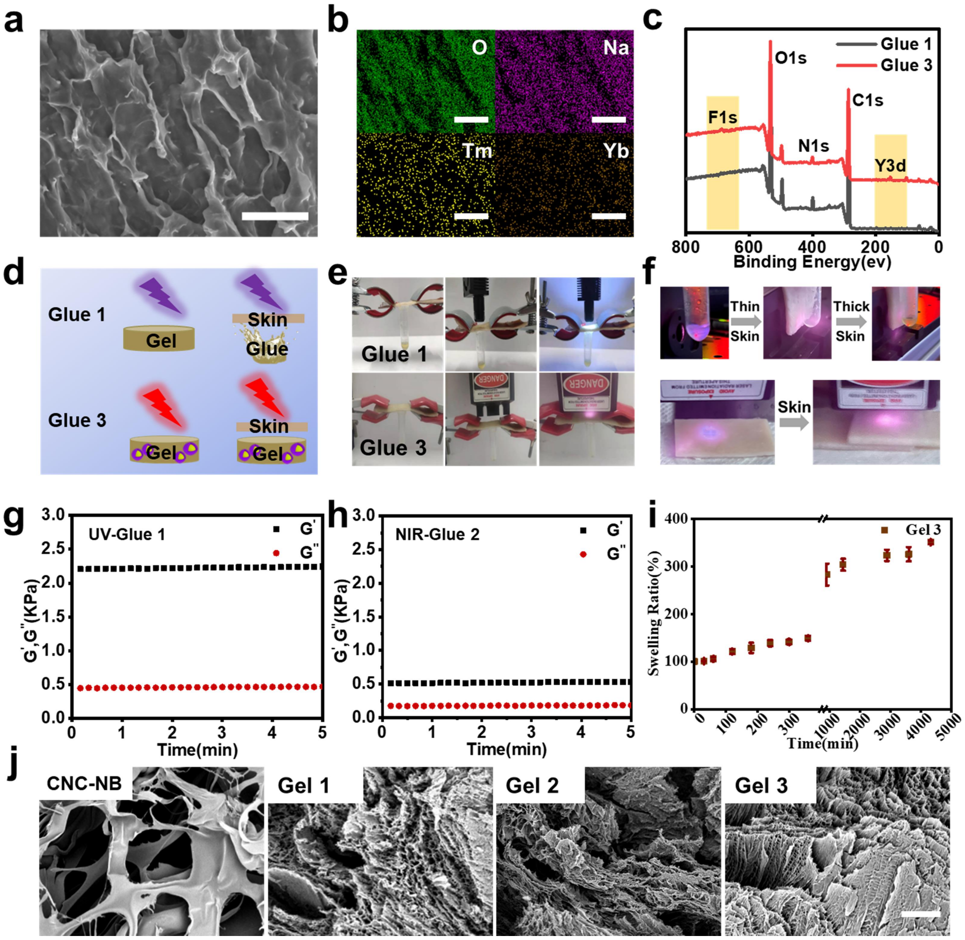

Figure S6. Fabrication and characterization of smart bio-glues and hydrogels. (a) SEM images and (b) EELS mapping images of Glue 3. (Scale bar: 25 μm) (c) XPS survey spectra of Glue 1 and Glue 3. (d) Schematic illustration of light-triggered hydrogels formation with and without a pigskin-covered. (e) Photographs of glues and hydrogels with pig skin-covered under UV and NIR irradiation. (f) Photographs of glues under thin and thick pigskin-covered under NIR irradiation. Dynamic storage modulus (G′) and loss modulus (G″) at different time points of (g) Glue 1 after UV irradiation and (h) Glue 2 after NIR irradiation. (i) Swelling ratios of Glue 3 after NIR irradiation. (j) SEM images of NB-CNC, Glue 1 after UV irradiation, Glue 2, and Glue 3 after NIR irradiation, respectively. (Scale bar: 100 μm)

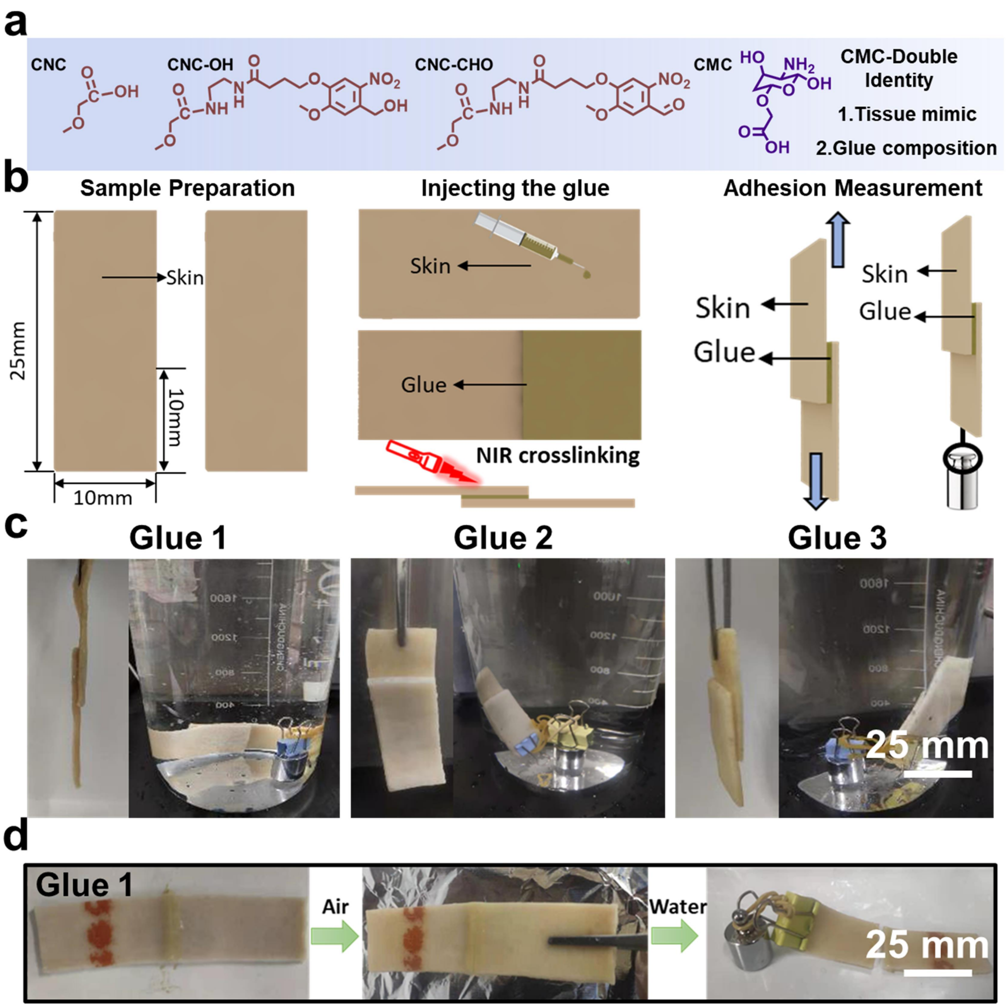

Figure S7. Calculation and adhesion capability testing of smart bio-glues. (a) Typical chemical functionality of CNC, CNC-OH, CNC-CHO and CMC (CMC has a double identity including tissue mimic and glue composition). (b) Schematic illustration of a pigskin adhesion measuring model. (c) Optical images of adhesion performance of Glue 1, Glue 2, Glue 3. (d) Optical images of adhesion performance of Glue 1 under UV irradiation.

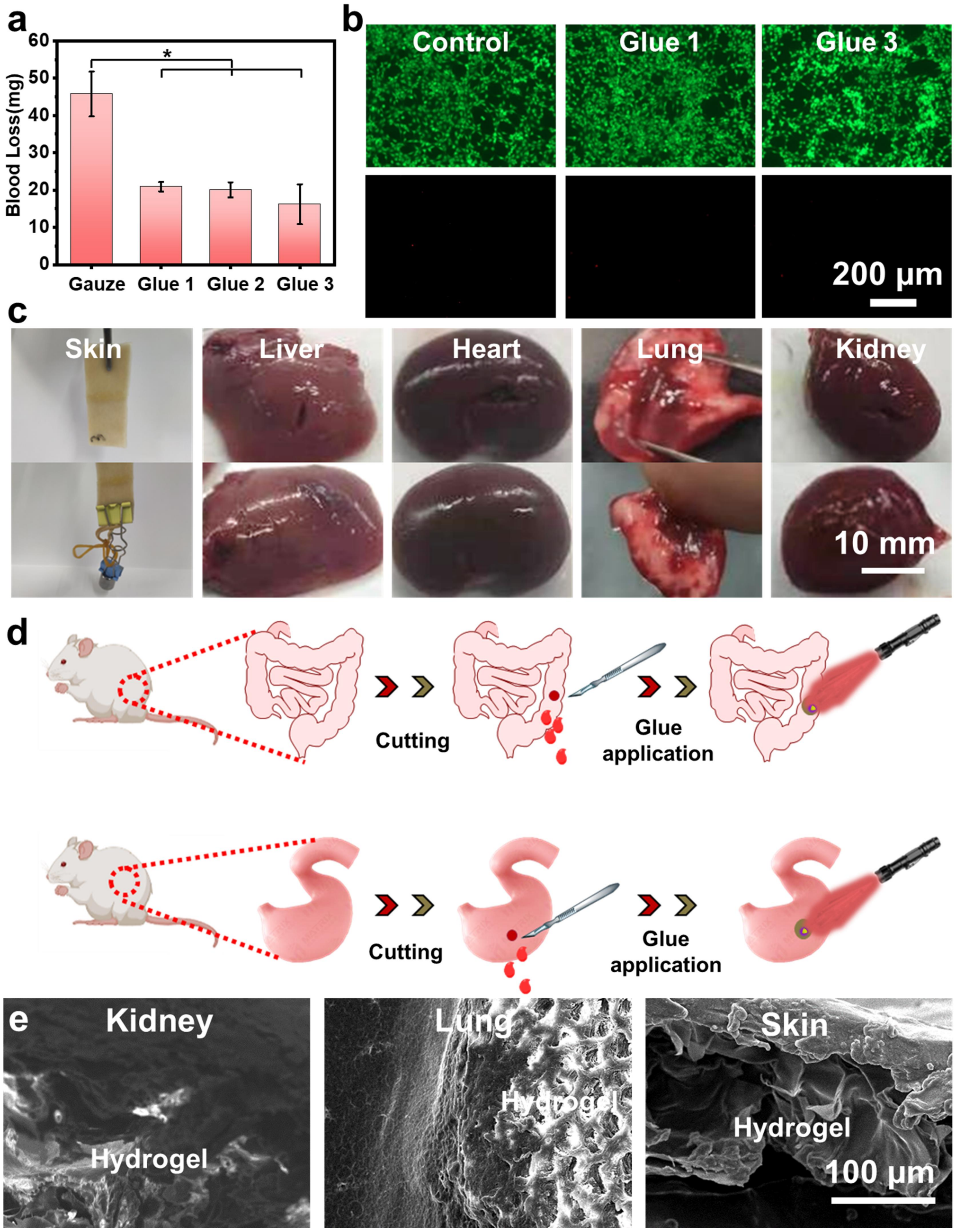

Figure S8. Biocompatibility and bioadhesion applications of smart bio-glues. (a) *In vivo* hemostasis evaluation performed by recording blood loss in dry state. (b) *In vitro* cytocompatibility of the glues in Live/Dead staining assay of NIH 3T3 cells. (c) Optical images of tissue adhesion in various perforate damaged tissues. (d) Schematic representation of glues application in gastric and intestinal perforation repair. (e)

Representative SEM images showing the interfaces between Glue 3 and different tissues. *$p < 0.05$, **$p < 0.01$, ***$p < 0.001$.

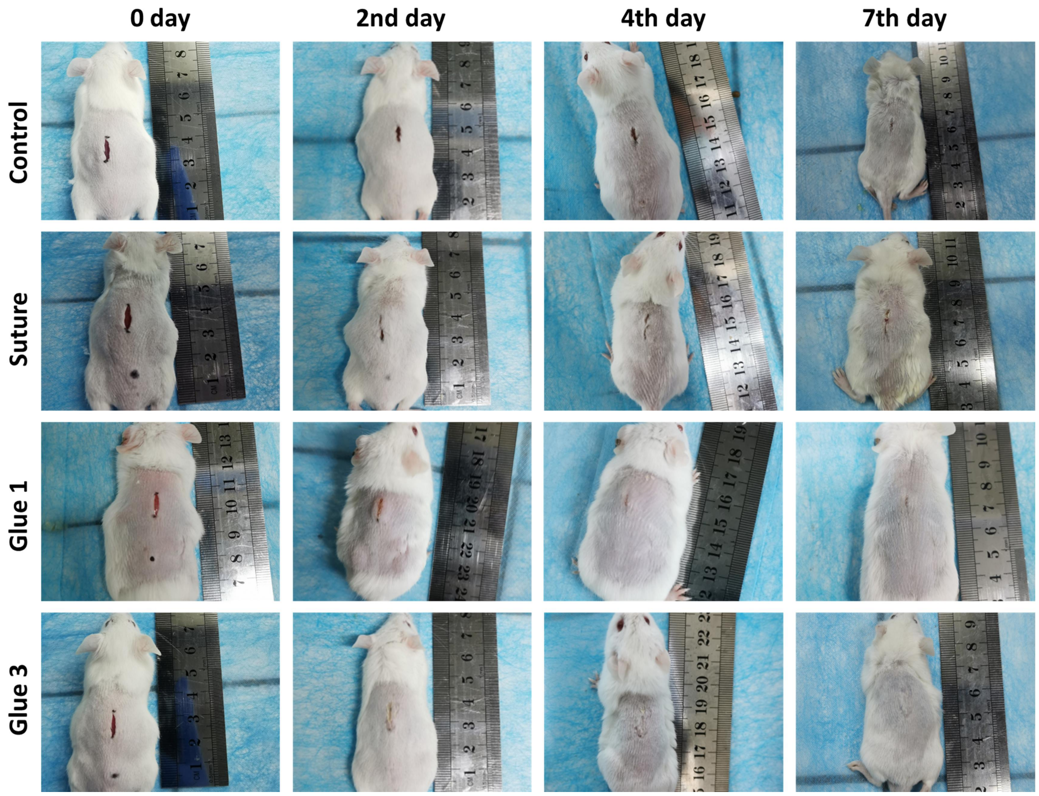

Figure S9. Representative optical images of smart bio-glues for external wound healing in a rat model.

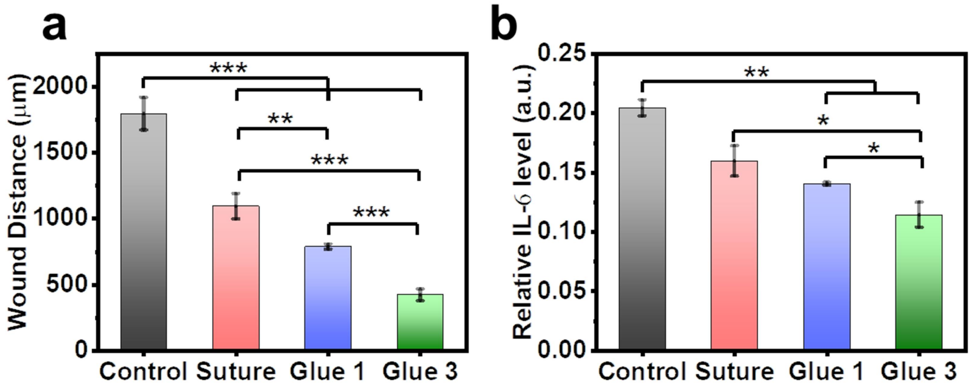

Figure S10. (a) Relative wound distance after 7 days with different treatment. (b) Relative level of inflammatory responses after 7 days with IL-6 (n=5). *$p < 0.05$, **$p < 0.01$, ***$p < 0.001$.

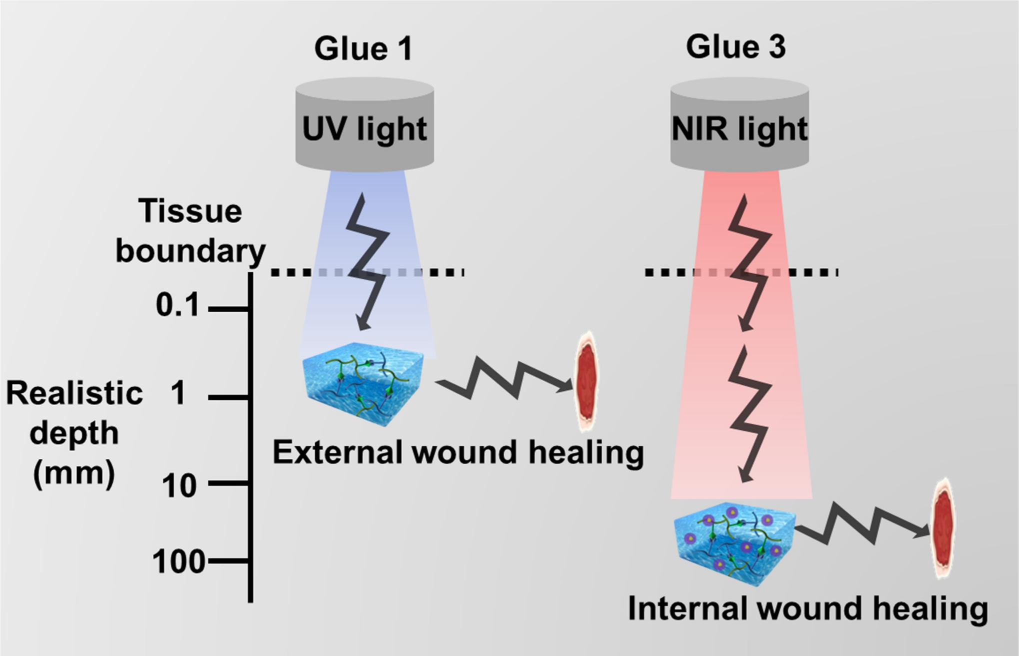

Figure S11. Schematic illustration of the mechanism of the smart bio-glues for wound healing, where Glue 1 (UV light) only can be used for external wound healing, while Glue 3 (NIR light) could be applied to external and internal wound healing.

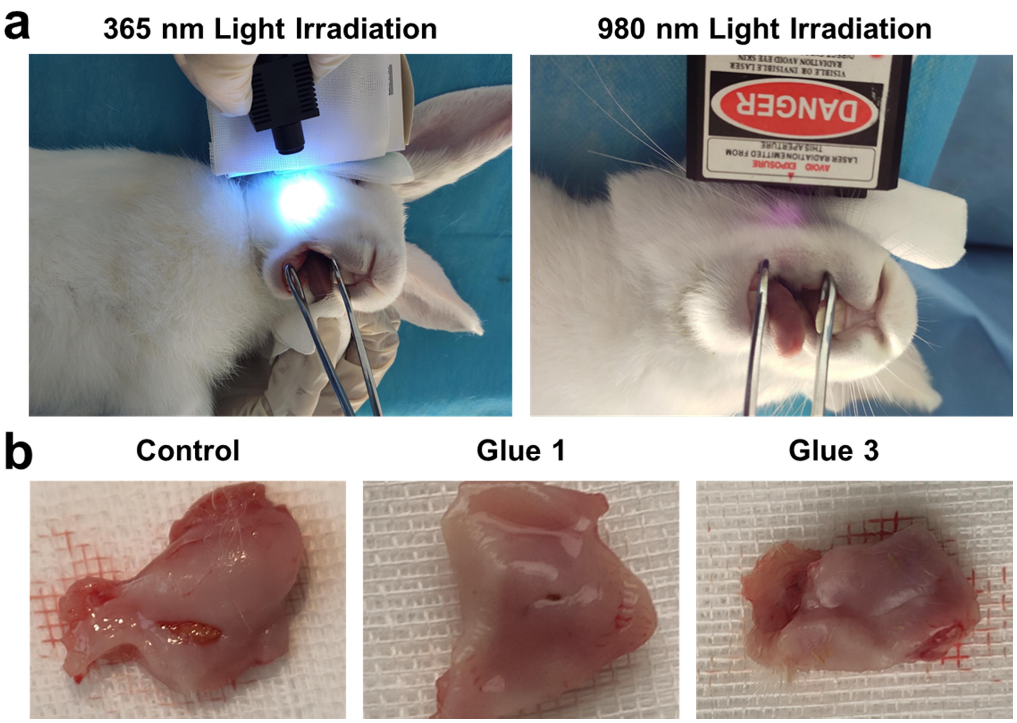

Figure S12. (a) Representative optical images of smart bio-glues for internal wound healing in a rabbit model and (b) obtained wound samples for further analysis.

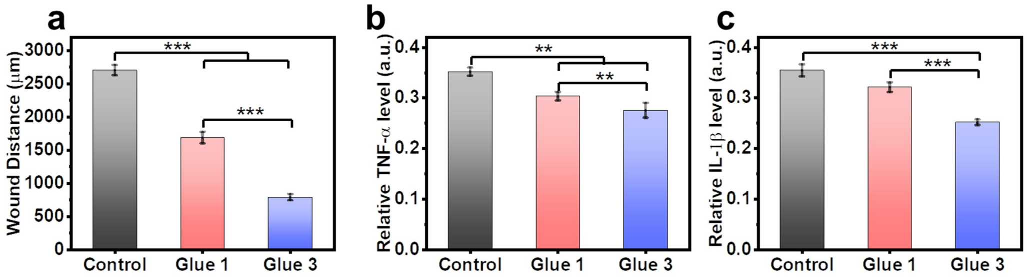

Figure S13. (a) Relative wound distance after 3 days with different treatment. (b) Relative level of inflammatory responses after 3 days with TNF-α and IL-1β (n=5). $*p < 0.05$, $**p < 0.01$, $***p < 0.001$.

Table S1. Summary of the formula of Glue i (i = 1 to 3).

Number	Composition	H_2O	UCNPs (w/v)	Sol-Gel condition
Glue 1	6mg NB-CNC+6mg CMC	200 μL	0%	UV-2 min
Glue 2	6mg NB-CNC+6mg CMC	200 μL	0.5%	NIR-2 min
Glue 3	6mg NB-CNC+6mg CMC	200 μL	1%	NIR-2 min

Movie. S1. Smart bio-glues for internal wound healing in a rabbit model under UV light irradiation.
Movie. S2. Smart bio-glues for internal wound healing in a rabbit model under NIR light irradiation.
